# Experiences with a system for signal- and data-processing, together with on-line variance reduction, in continuous-flow analysis

**DOI:** 10.1155/S1463924684000304

**Published:** 1984

**Authors:** R. Maassen, J. C. Smit, H. Baadenhuijsen

**Affiliations:** Department of Internal Medicine Division of Clinical Chemistry St. Radboud University Hospital Geert Grooteplein Zuid 8 Nijmegen NL6500 HB The Netherlands


Journal of Automatic Chemistry, Volume 6, Number 3 (July–September 1984), pages 158–160

### Experiences with a system for signal- and data-processing, together with on-line variance reduction, in continuous-flow analysis

**R. Maassen, J. C. Smit and H. Baadenhuijsen**

*Department of Internal Medicine, Division of Clinical Chemistry, St. Radboud University Hospital, Geert Grooteplein Zuid 8, NL6500 HB, Nijmegen, The Netherlands*

### Introduction

For the investigation of the performance of a mathematical filter for variance reduction, a previously described [1] computer program for peak recognition and data-processing for a continuous-flow system was adapted to accomodate subroutines for base-line drift compensation and between-run variance reduction. The subroutine for between-run variance reduction was based on the ideas and algorithms reported by Jansen and Bonants [2].

Whereas for most components determined with continuous-flow analysis a within-run coefficient of variation (CV) of about 1–1·5% can be obtained, which compares favourably with newer analytical techniques like centrifugal analysis, the between-run CV of 2·5–3·5% was judged to be too large. From the work of Jansen and Bonants it can be concluded that between-run variance reductions between 40 and 70% are obtainable by applying a digital filtering technique to the information content of control sera placed and analysed in every run.

### Hardware

The system (see figure 1) was configured around a 48 kbyte ITT 2020, equipped with a 9 in monitor, a floppy disk drive (Apple II) and a matrix printer (Epson TX-80). The ITT 2020 also has an Autostart ROM, a printer interface (Epson, slot 1), an interrupt timer (CCS 7440A, slot 2), an analogue/digital (A/D) converter (CCS 7470A, slot 3), an amplifier board (home-made, slot 4) and a disk drive controller (Apple II, slot 6).

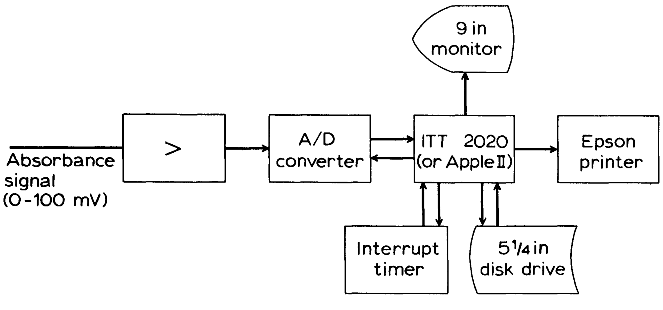

*Figure 1. Block diagram of the electronic processing system.*

### Memory use

As compared to the old situation [1], the size of the program was increased to such an extent that it was necessary to change the memory organization in order to prevent overwriting of variables, array-contents, strings and pointers by the data from the A/D converter. Figure 2 shows the situation before and after the extension of the program. Previously, the data from the A/D converter were dumped into memory locations between 16384 and 32760 by the 6502 Assembler routine. The space left was just large enough for the main Basic program. In the new situation the Assembler routine is changed in such a way that the data are dumped into an 8192 byte block; the starting address has to be a multiple of 8192 and in this case 24576 was chosen. When the highest address has been reached the routine automatically restarts at address 24576. The Basic program has been adapted to account for these changes. In order to save some more memory, and to be able to process an unlimited number of analyser peaks, a subroutine which enables reuse of arrays has been incorporated. This subroutine transfers the latest 30 results into array positions 1 to 30 and resets all the pointers involved.

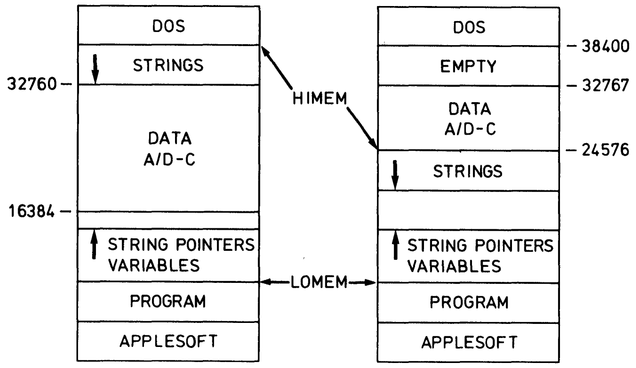

*Figure 2. Memory-map of the 48 kbyte ITT 2020 microcomputer before (left) and after (right) the extension of the program.*

### Base-line drift compensation

In some continuous-flow applications base-line drift can be a considerable problem, as was the case with the system for measuring serum creatinin which the authors used for this investigation. Assuming the drift between two successive calibration series to be linear in time, a subroutine for drift correction was written. After each calibration series, the samples of the previous run are corrected for drift using the old and new base-line values (calculated by linear regression analysis).

### Between-run variance reduction

It is well known that in analytical processes a significant between-batch variance can exist. It seems that some kind of impulse-like disturbance occurs each time a new run is analysed. In order to reduce this between-run variance, and thus also the overall variance, a mathematical filter was developed [2]. Using relative measurements (deviations from the target values) of control sera, placed in every run, this filter estimates the relative deviation for the run in question from a weighted preceding estimate, plus the weighted mean, in the following recursive algorithm:

$$S_r = \alpha S_{r-1} + (1-\alpha) D_r$$

(where $\alpha = \exp(-1/T)$, T is a filter time constant, $S_r$ is the estimate of the relative deviation in run $r$, $S_{r-1}$ is the same estimate for the previous run $r-1$, and $D_r$ is the mean relative deviation of the control sera from their target values in run $r$). The estimate $S_r$ is used to correct the remaining samples in run $r$ according to:

$$Z_{cir} = Z_{ir} - Z_{ir} \times S_r$$

(where $Z_{cir}$ is the corrected value for sample $i$ of run $r$, and $Z_{ir}$ is the measured value for sample $i$ of run $r$). For a complete theoretical description and evaluation of the model see Jansen and Bonants [2].

To assess whether a significant variance reduction was obtainable, additional control sera, which were not used for the estimation of the varying process fluctuations, were placed in the runs analysed. A subroutine for this digital filter was added to the program, together with two statistical quality-assessment tests.

### Statistical tests

Before any filter calculations or corrections were made, the range covered by the highest and the lowest control serum (relative values) was tested with a $\chi^2$-test to determine the quality of the particular run. In addition, the bias of the mean of the controls with the target values (relatively taken) was tested with the Student $t$-test. The percentage variance reduction ($VR$) is defined as:

$$VR = (1 - S_c^2/S_n^2) * 100\%$$

(where $S_c^2$ is the variance of the corrected results, and $S_n^2$ is the variance of the uncorrected results).

### Logical design

Figure 3 is a flow diagram of the program.

### Results and discussion

With this program 158 runs for the determination of serum creatinine (alkaline picrate method) were analysed and the results stored on floppy disk. Each run consisted of four aqueous creatinine standards, nine control sera, three aqueous control standards and two patient sera. Each sample cup was sealed with Parafilm in order to avoid evaporation. A total of nine runs was

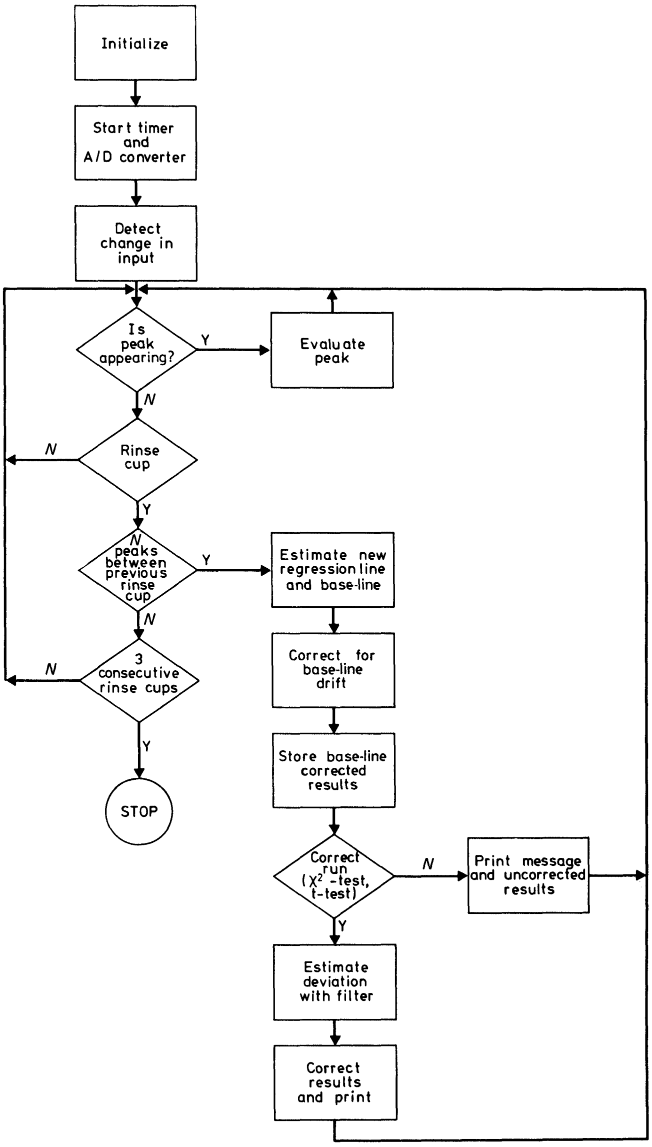

*Figure 3. Flow chart of the program. At the third decision diagram from above, 'N' stands for the number of calibration specimens in the calibration series.*

*Table 1. Composition and overall results of the 158 runs analysed.*

| Position, material | Origin | Mean (μmol/l) | Variance | Coefficient of variation |
|---|---|---|---|---|
| 1 serum F | bovine | 217·2 | 15·58 | 1·82 |
| 2 patient serum | | | | |
| 3 standard (50 μmol/l) | aqueous | 50·4 | 2·64 | 3·23 |
| 4 serum D | horse | 103·2 | 4·05 | 1·95 |
| 5 serum F | 218·6 | 14·18 | 1·73 | |
| 6 standard (150 μmol/l) | | 147·8 | 5·34 | 1·56 |
| 7 serum A | human | 94·1 | 4·16 | 2·17 |
| 8 patient serum | | | | |
| 9 serum C | human | 143·3 | 7·21 | 1·87 |
| 10 serum E | bovine | 63·2 | 2·65 | 2·58 |
| 11 serum B | human | 227·2 | 9·28 | 1·34 |
| 12 standard (200 μmol/l) | | 197·9 | 7·18 | 1·35 |
| 13 serum D | | 104·0 | 3·52 | 1·80 |
| 14 serum E | | 63·1 | 2·48 | 2·49 |

*Table 2. Mean percentage variance reduction with use of two or three control sera in the estimating filter. (Time constant T of the filter T→0.)*

| Position/identity<br>Test sera | 1<br>F | 4<br>D | 5<br>F | 7<br>A | 9<br>C | 10<br>E | 11<br>B | 13<br>D | 14<br>E | Mean %<br>*VR* |
|---|---|---|---|---|---|---|---|---|---|---|
| Position/identity<br>Control sera | | | | | | | | | | |
| 4, 11/D, B | | | | | | | | | | 38·1 |
| 1, 14/F, E | | | | | | | | | | 20·5 |
| 5, 10/F, E | | | | | | | | | | 22·7 |
| 7, 13/A, D | | | | | | | | | | 20·6 |
| 7, 9, 11/A, C, B | 45·9 | 24·3 | 50·6 | | | 35·6 | | 35·6 | 33·7 | 37·6 |
| 4, 5, 10/D, F, E | 35·7 | | | 54·5 | 62·6 | | −14·5 | 15·5 | 25·3 | 29·8 |
| 4, 7, 11/D, A, B | 55·1 | | 62·3 | | 61·3 | 27·4 | | 26·9 | 24·1 | 42·9 |
| 3, 6, 12/stand. | 40·2 | 59·4 | 40·7 | 27·1 | 18·7 | 2·8 | −6·5 | 1·5 | −4·6 | 19·9 |
| 1, 13, 14/F, D, E | | 13·9 | 29·6 | 48·6 | 45·3 | 26·2 | −22·1 | | | 23·6 |
| 1, 9, 14/F, C, E | | 20·3 | 41·1 | 56·9 | | 33·3 | −26·2 | 45·9 | | 28·6 |
| Mean percentage *VR* for individual sera | 44·2 | 29·5 | 44·9 | 46·8 | 47·0 | 25·1 | −17·3 | 25·1 | 19·6 | |

excluded from the calculations because of excessive outliers. With the remaining 149 runs, all calculations were carried out using a separate evaluation program. The authors investigated whether there are favourable positions within the run for control sera, whether aqueous control standards are suitable for use in the variance reduction estimation and what the best choice is for the filter time constant, T.

Table 1 shows means and variances of the sera and aqueous controls and the different locations in which they were used. It appears that two pairs of equal control sera do not necessarily have the same grand mean. Serum F, on positions 1 and 5, shows respective means of 217·2 and 218·6—a statistically significant difference. The same holds for serum D, on positions 4 and 13, with respective means of 103·2 and 104·0. Serum E, on the other hand, shows exactly the same grand mean on different locations (10 and 14, with values of 63·2 and 63·1). It cannot, therefore, be concluded that there is a trend-like increase of the results within a single run.

Table 2 gives the results of the variance reduction achieved with different combinations of two and three control sera in the estimating filter. The mean percentage variance reduction has always been calculated for those sera that were not used in the filter algorithm. Depending on the kind of serum, variance reductions of 20–50% were obtained when applying this digital-filtering technique.

As shown by the values for the mean percentage variance reduction with the various control serum combinations, there is no particular advantage in using three rather than two control sera. So for all practical purposes two control sera suffice. From the individual variance reduction figures, given in table 2, it is not possible to conclude that particular positions in the run are to be preferred. The slightly better performance of combination 4, 5, 10, as compared with 1, 13, 14, in both instances measured on the variance reduction on position 7, 9, 11, might lead to the conclusion that control sera placed at the beginning or end of a run do worse than those in the middle of a run. However, this conclusion seems not to be valid when the combination 4, 5, 10 and 1, 9, 14 are compared on the variance reduction on positions 7, 11, 13. From the remarkable results of serum B it can be seen that the kind of serum influences the variance reduction performance. This is also illustrated by the mean variance reduction obtained for each individual serum. Serums F (positions 1 and 5), A (position 7) and C (position 9) all show roughly the same mean variance reduction—about 45%. Serums D (positions 4 and 13) and E show an intermediate performance.

The performance of the filter depends on the choice of the filter time constant, T. It was noted that varying the value of T between 0 and 1 produced good results (*VR* range 33–40%) with an optimum for T = 0·5. One of the highest-ranking requirements in the initial design of this microcomputer-based continuous-flow read-out interface [1] was the total flexibility of the system: no restrictions whatsoever were imposed on the run length. This could be achieved by letting the program look for a possible calibration series placed somewhere between the patient serum specimens. It was realized that the reservation of positions for quality-control purposes would substantially reduce this flexibility. Since the results of the experiments do not indicate any preferred or better excluded positions within a run, it was decided to stick to the original flexibility requirement by purposely linking the positions of the control sera to those of the calibration standards.

Recalling the logical procedure which was used in the program to recognize a calibration series, the procedure is now as follows. After having detected a series of five analyser peaks, inserted between two rinse-cups, the program carries out linear regression analyses on these peaks. After finding that the linear regression coefficient surpasses the value of 0·995, the program recognizes these five peaks as a calibration series. Thereafter, the last preceeding and first succeeding peak are taken as control sera and these are labelled with their respective target values in data statements at the beginning of the program.

This means that the original flexibility of the read-out device has been preserved together with an average between-run variance reduction of 30%–40%.

### References

1. Baadenhuijsen, H. and Zelders, Th., *Journal of Automatic Chemistry*, **5** (1983), 18.
2. Jansen, R. T. P. and Bonants, P. J. M., *Annals of Clinical Biochemistry*, **20** (1983), 174.

